# Morphological and molecular identification of third-stage larvae of *Anisakis typica* (Nematoda: Anisakidae) from Red Sea coral trout, *Plectropomus areolatus*

**DOI:** 10.1007/s00436-022-07776-1

**Published:** 2023-01-18

**Authors:** Nesma Abbas Mostafa, Fathy Abdel-Ghaffar, Hamed Omar Fayed, Ayat Adel Hassan

**Affiliations:** Zoology Department, Faculty of Science, Cairo University, Giza, Egypt

**Keywords:** *Plectropomus areolatus*, Morphology, *Anisakis typica*, Molecular analysis, Egypt

## Abstract

Anisakidosis is a foodborne zoonotic infection induced by members of the family Anisakidae via the consumption of raw or undercooked fish such as sushi and sashimi. Identifying anisakid larval species is critical for the epidemiology and diagnosis of diseases caused by them. This study aimed at identifying *Anisakis* larvae collected from marine fish in Egyptian waters based on morphological characteristics and molecular analysis. Thirty marine fish coral trout, *Plectropomus areolatus*, were collected from Hurghada, Red Sea, Egypt, to investigate larval nematodes of the genus *Anisakis*. The larvae were detected encapsulated in the peritoneal cavity and muscle of the fish host. This examination revealed that anisakid larvae naturally infected 19 fish specimens with a prevalence of 63.33% and a mean intensity of 4.1 ± 0.40. Most of them (68 larvae: 71.57%) were found in the musculature. Morphological and morphometric analyses using light and scanning electron microscopy revealed a head region with a prominent boring tooth, inconspicuous lips, and a characteristic protruded cylindrical mucron. All larvae in this study possessed the same morphology as *Anisakis* Larval type I. Molecular analysis based on ITS region using maximum likelihood and Bayesian phylogenetic methods confirmed them as *Anisakis typica*. This is the first study to identify *A. typica* larvae from the commercial fish coral trout *P. areolatus* in Egyptian waters using morphological and molecular methods.

## Introduction

Fish-borne zoonotic nematodes are a significant public health problem worldwide (Chai et al. 2005; Mattiucci et al. 2018; Bao et al. 2019). Anisakid nematodes are cosmopolitan parasites of marine organisms, with marine mammals serving as their definitive hosts, and the third-stage larvae infect marine fishes as paratenic hosts (Nieuwenhuizen and Lopata 2013; Pozio 2013). Anisakidosis is a zoonotic disease acquired through eating raw or insufficiently cooked fish infected with third-stage larvae of the genera *Anisakis*, *Pseudoterranova*, and *Contracaecum* (Mattiucci and Nascetti 2008; Bao et al. 2017; Shamsi et al. 2018). Infections by anisakid larvae (L3) are widespread in Europe and eastern Asia, commonly in Japan, Peru, and Chile, as a result of increasing the consumption of raw or undercooked fish and seafood (Chai et al. 2005; Sohn et al. 2015; Eiras et al. 2018; Martínez-Rojas et al. 2021). In humans, these parasites can cause severe gastroenteritis (Shamsi and Butcher 2011; Baptista-Fernandes et al. 2017). They also contribute to allergic reactions (Audicana and Kennedy 2008; Lopata and Lehrer 2009; Jabbar et al. 2012). These reactions are caused by chemical compounds found in fish meat, which are produced by the parasite (Pozio 2013; Ivanovic et al. 2017; Kochanowski et al. 2020).

The first report of infection by *Anisakis* larvae in humans documented by Van Thiel et al. (1960) raised awareness of fish-borne parasitic diseases. These nematodes penetrate the stomach wall and cause gastrointestinal symptoms, including stomach pain, vomiting, and nausea (Smith and Wootten 1978; Valls et al. 2005; Ivanović et al. 2017; Mattiucci et al. 2018). Furthermore, *Anisakis* infection has been linked to an increased risk of gastrointestinal cancer or tumors (Garcia-perez et al. 2015; Corcuera et al. 2018).

Anisakid larvae can be identified at the genus level by the morphological features of anterior and posterior regions; these larvae are further divided into four groups, types I–IV, based on the length of the ventriculus and the presence or lack of a tail spine (mucron). *Anisakis* type I larvae have a longer ventriculus and a distinct mucron, including *A*. *simplex* s.s., *A*. *pegreffii*, *A*. *typica*, *A*. *ziphidarum*, *A*. *berlandi*, and *A*. *nascettii*, while types II–IV larvae have a shorter ventriculus and no mucron, including *A*. *physeteris*, *A*. *brevispiculata*, and *A*. *paggiae* respectively (Murata et al. 2011; Mattiucci et al. 2018; Shamsi 2021).

Specific morphological identification of L3 anisakid larvae is relatively challenging due to the low development of the organs at this stage and the absence of remarkable features (Tunya et al. 2020). Generally, the difficulty of identifying nematodes depends on several parameters, such as the small size of the worms, the great diversity of nematodes found in a sample, and the absence of specific morphological traits (Farjallah et al. 2008; Seesao et al. 2017). Furthermore, they are retrieved from frozen fish or patient organs and tissues (Dick et al. 1991; Abou-Rahma et al. 2016). Therefore, molecular techniques, such as DNA sequencing, are the most reliable methods to identify anisakid larvae (Lin et al. 2007; Tunya et al. 2020). Several studies have demonstrated the utility of nuclear and mitochondrial DNA markers such as large ribosomal DNA (28S), internal transcribed spacer (ITS), and cytochrome c oxidase subunit 1 (cox1) and subunit 2 (cox2) for the identification of ascaridoid worms (Li et al. 2017; Zhang et al. 2018; Guo et al. 2021). In Egypt, some previous studies recorded *Anisakis* spp. from different marine fish exhibiting various prevalences reached 76.7% of *Anisakis* sp. (type I) in European sea bass *Dicentrarchus labrax* (Morsy et al. 2012), 35% of *Anisakis* sp. (type II) in greater lizard fish *Saurida undosquamis* (Morsy et al. 2015), 36.6% of *Anisakis simplex* s.s. from European Hake *Merluccius merluccius lessepsianus* (Abou-Rahma et al. 2016), 19.05% and 42.86) of *Anisakis* sp. (type I) in smoked herring and frozen mackerel fish (Arafa et al. 2019), 87.1% and 83.3% of *Anisakis simplex* s.s. in Atlantic herring and Mediterranean horse mackerel, whereas 42.8% of *Anisakis typica* in Atlantic mackerel (Mostafa et al. 2020).

The study aimed to contribute to the epidemiology and molecular identification of *Anisakis* spp. infecting commercially important fish in Egypt.

## Materials and methods

The current study was performed following guidelines approved by the Cairo University Institutional Animal Care and Use Committee (CU-IACUC) and approved under the relevant document (No. CU/I/F/32/19).

A total of 30 coral trout, *P. areolatus* (F: Serranidae), were purchased from local fish markets in Hurghada city, Red Sea. Fish specimens were dissected and the body cavity, digestive tract, visceral organs examined for nematode parasites. The musculature was sliced into thin slivers (1.0–2.0-mm thick) and then visually inspected for parasites under white light. Larvae were removed from the surrounding host tissues with the aid of a stereomicroscope, noting the site of infection then washed in physiological saline, counted and preserved in 70% ethanol until use.

Nematodes were cleared in lactophenol for morphological studies (Pritchard and Kruse 1982). The total number of larvae were analyzed (*N* = 25), and the number of measured larvae were (*N* = 5) for every parameter. The identification was based on the main characteristics of larval anisakids, such as anteriorly located boring tooth or lips, the length of the ventriculus, the postanal tail’s shape, and the presence or absence of a terminal mucron (Pinto et al. 1994; Rocka 2004). All body measurements were taken with ocular micrometers and photomicrographic images were obtained with a LEICA DM 750 microscope. Scanning electron microscopy (SEM) was used to examine nematode larvae that had been fixed in a glutaraldehyde solution of 2.5%. After 24 h, samples were post-fixed in phosphate buffer containing 1% osmium tetroxide (OsO4) for 24 h, dehydrated through a graded ethanol series (50%, 60%, 70%, 80%, 90%, and 100%), and dried at 30 °C for 30 min using a critical point drier (LEICA, EM CPD300). Dried specimens were mounted on aluminum stubs with carbon tape, coated with gold, and examined with a JEOL JSM-5200 SEM (Tokyo, Japan) at an accelerating voltage 25 kV (Guo et al. 2014).

For molecular studies, Genomic DNA was extracted from individual larvae (*N* = 25) after being preserved in 70% ethanol using a QIAamp® DNA Mini Kit (Qiagen) following the manufacturer’s protocol. PCR amplification of the internal transcribed spacer (ITS1-5.8S-ITS2) region of ribosomal DNA (rDNA) used two universal primers NC5 (forward; 5′-GTAGGTGAACCTGCGGAAGGATCATT-3′ and NC2 (reverse; 5′- TTAGTTTCTTTTCCTCCGCT-3′) (Zhu et al. 1998). PCR reactions were performed in a total volume of 50 μl as follows: 25 μl PCR Super-Mix (Genetech) containing dNTP, MgCl2, buffer, and Taq-polymerase,1 µl of 10 Pmol of both forward and reverse primers and 3 μl parasite genomic DNA, and then it completed by 20 μl of nuclease free water. Thermocycling conditions involved an initial denaturation step at 94 °C for 5 min, then 35 cycles of denaturation step at 94 °C for 30 s, primer annealing at 58 °C for 30 s, extension at 72 °C for 30 s, and a final extension at 72 °C for 7 min (Thermal Cycler, Model FTC3/20 (TC-3000X, TECHNE, Bibby scientific, and United Kingdom) according to Costa et al. (2018) with some modifications.

PCR products were analyzed by gel electrophoresis and visualized using a UV transilluminator (Cedex 1, France). Stained DNA fragments were photographed using a gel documentation analysis system. PCR products were purified using a gel purification kit (Genedirex. Inc) and sequenced using an automated sequencer, ABI PRISM model 377 version 3.3.1 (Clinilab, Egypt). The nucleotide sequence obtained in this study was deposited in GenBank under accession number OM371077. BLAST searches were performed at the National Center for Biotechnology Information (NCBI) database (http:// www.ncbi.nlm. nih.gov) to find sequence similarities. The query sequence plus those retrieved from GenBank were aligned using Bioedit version 3.3.19.0, and phylogenetic trees were constructed using maximum likelihood method based on Kimura 2-parameter model by the MEGA software version 11.0.10 (Tamura et al. 2021) with bootstrapping of 1000 replications and a Bayesian method in BEAST software version v1.10.4 (Suchard et al. 2018). *Procamallanus fulvidraconis* (DQ076698) was used as an outgroup.

## Results and discussion

Compared to earlier investigations in Egypt on this genus, 95 nematode specimens were found in 19 out of 30 *P*. *areolatus* investigated (63.33%), as indicated in Table 1. All obtained samples correspond to *Anisakis* L3. The larvae were found encapsulated in the fish’s body cavity and muscles. The mean larval intensity was 4.1 ± 0.4 (1–5 larvae/fish). Most of them were retrieved from the fish muscles (68 larvae: 71.57%), in contrast to the recent finding of Tunya et al. (2020), who isolated these *Anisakis* larvae primarily from the peritoneal cavity. Because humans frequently consume fish muscles, epidemiological concerns about the presence of larvae in muscles are significant, including human health, product safety, and ecological interest (Ayun et al. 2021). Furthermore, our larvae were only detected during visual inspection without incubation, in contrast to Shamsi and Suthar (2016), which reported that combining visual examination and incubation method was more effective in detecting the predominance of larvae. These parasites can emerge from internal organs and migrate to the flesh of the fish (Smith and Wootten 1975), which explains the higher prevalence of larvae in the muscles.

**Table 1:** Prevalence records of different *Anisakis* spp. in various marine fish in Egypt

Species	Fish host	Prevalence	Author
*Anisakis* sp. (type I)	European sea bass, *Dicentrarchus labrax*	(76.7%)	Morsy et al. (2012)
*Anisakis* sp. (type II)	greater lizard fish, *Saurida undosquamis*	(35%)	Morsy et al. (2015)
*Anisakis simplex* s.s	European Hake, *Merluccius merluccius lessepsianus*	(36.6%)	Abou-Rahma et al. (2016)
*Anisakis* sp. (type I)	Smoked herring *Clupea harengus*	(19.05%)	Arafa et al. (2019)
*Anisakis* sp. (type I)	Atlantic mackerel, *Scomber scombrus*	(42.86%)	Arafa et al. (2019)
*Anisakis simplex* s.s	Atlantic herring, *Clupea harengus*	(87.1%)	Mostafa et al. (2020)
*Anisakis simplex* s.s	Mediterranean horse mackerel, *Trachurus mediterraneus*	(83.3%)	Mostafa et al. (2020)
*Anisakis typica*	Atlantic mackerel, *Scomber scombrus*	(42.8%)	Mostafa et al. (2020)
*Anisakis typica*	coral trout, *Plectropomus areolatus*	(63.33%)	Present study

**Fig. 1:**
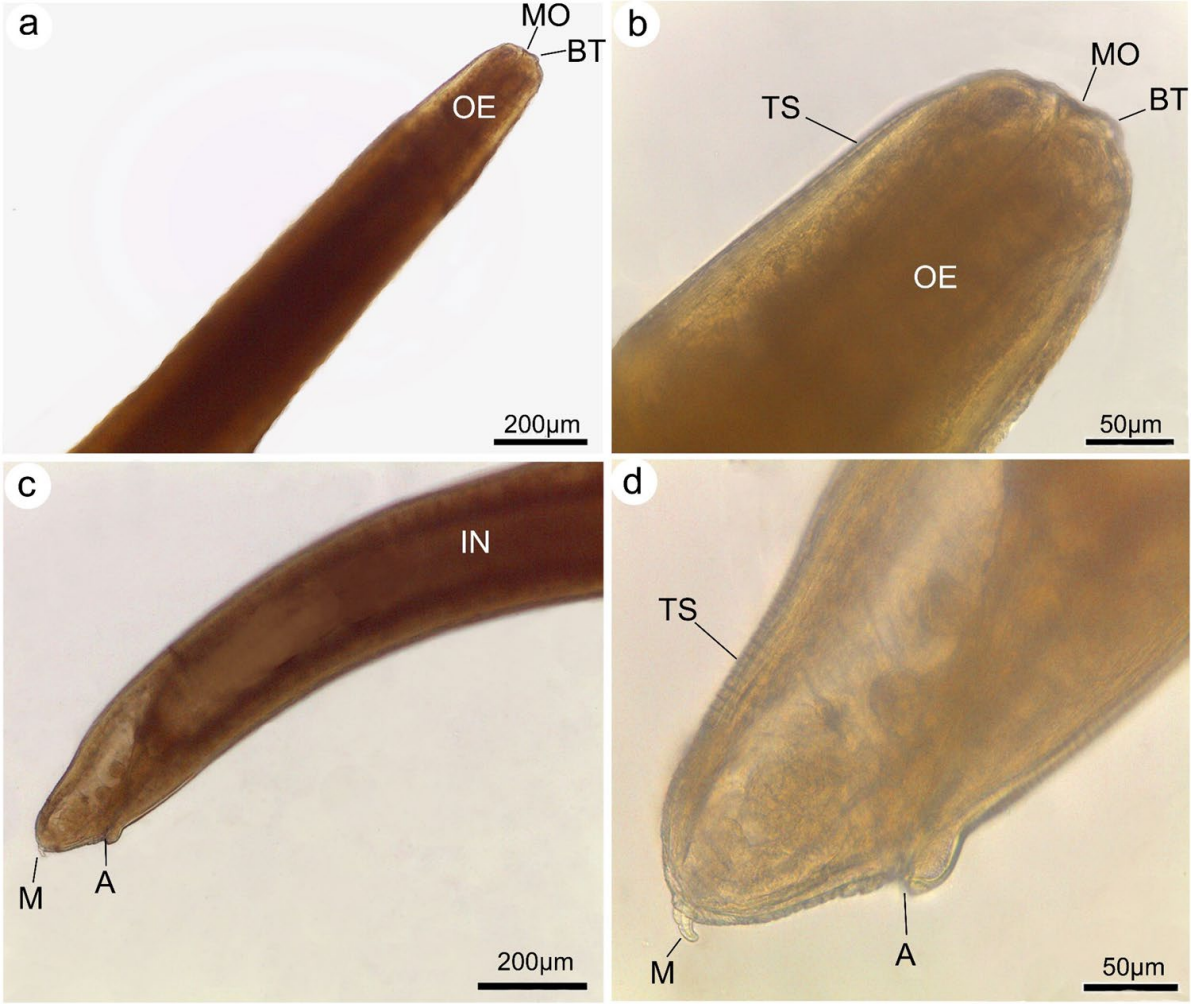
Photomicrographs of L3 *Anisakis typica* recovered from *P. areolatus* cleared in lactophenol. **a** Anterior region of worm showing mouth opening (MO) provided with boring tooth (BT) and long muscular esophagus (OS). **b** High magnification of anterior extremity indicate mouth opening (MO), boring tooth (BT), and esophagus (OS). **c** Posterior region showing intestine (IN) opens ventrally by anal opening (A) and ended with a cylindrical mucron (M). **d** High magnification of posterior extremity showing anal opening (A) and cylindrical mucron (M)

**Fig. 2:**
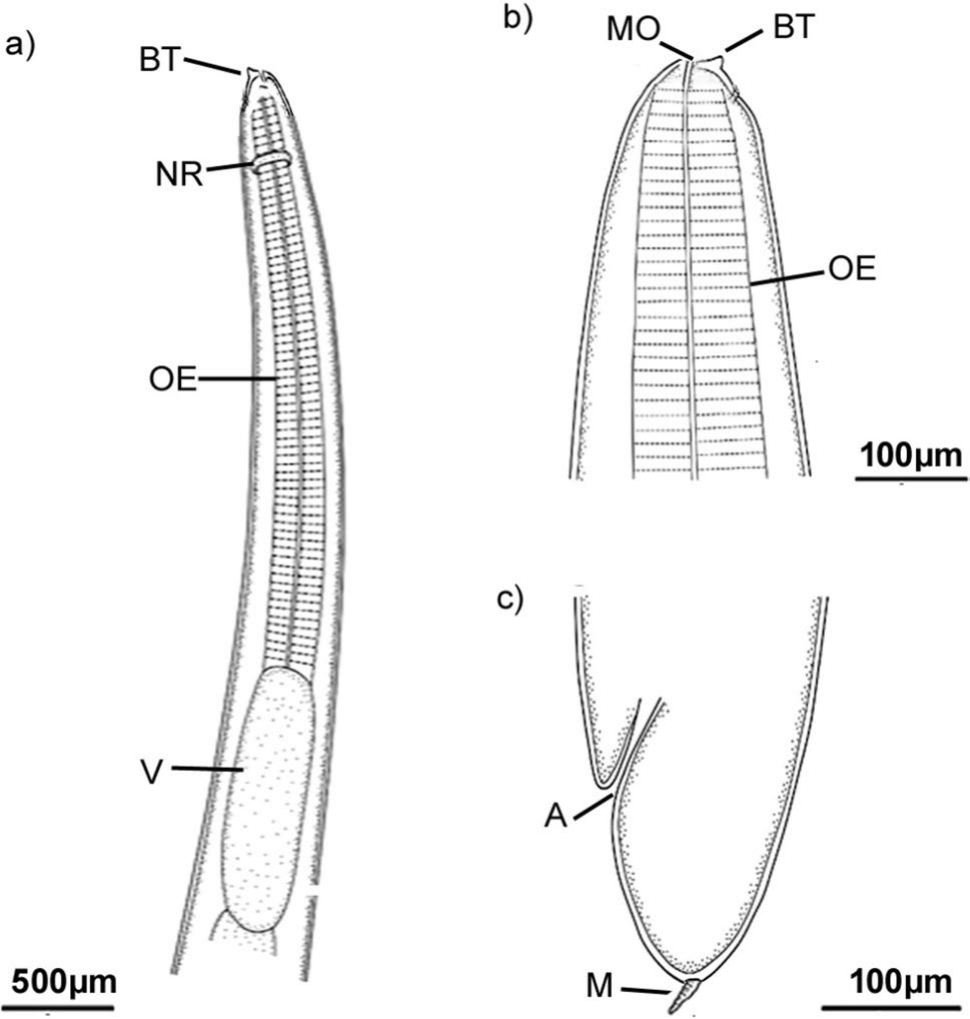
Drawings of L3 of *Anisakis typica* showing: **a, b** Anterior extremity with a boring tooth (BT), mouth opening (MO), esophagus (OE), nerve ring (NR), and ventriculus (V). **c** Posterior end showing anus (A) and rounded tail with a cylindrical mucron (M)

**Fig. 3:**
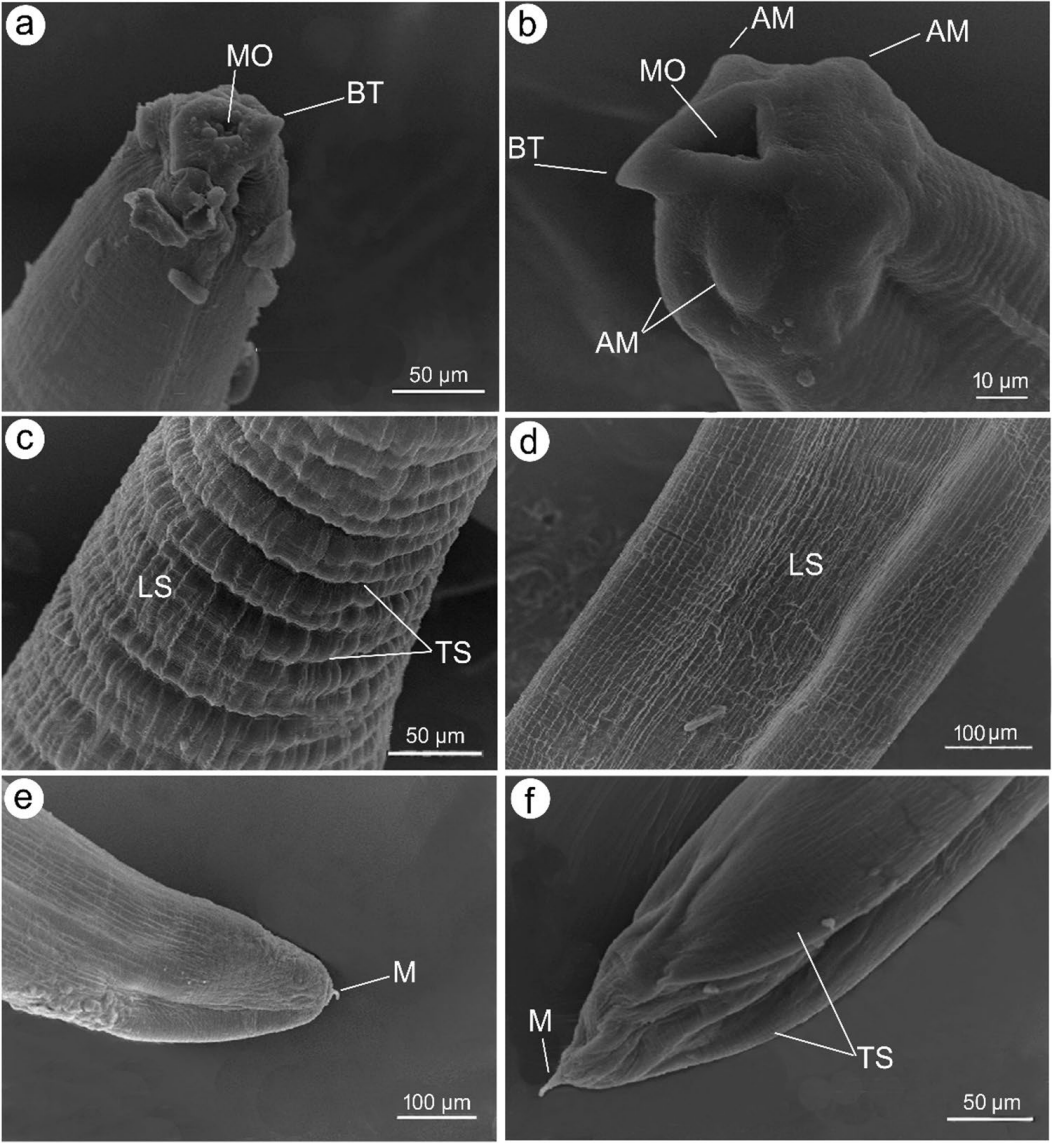
Scanning electron micrographs of L3 *Anisakis typica.* **a, b** Anterior extremity showing triangular mouth opening (MO), boring tooth (BT), and four amphids (AM). **c, d** Body cuticle showing longitudinal striations (LS) and transverse striations (TS). **e, f** Posterior extremity showing the characteristic mucron (M)

The morphological examination using a standard light microscope and SEM showed that the bodies of larvae were cylindrical, attenuated at both ends, and measured 18.1 ± 2.1 (16.58–19.68) mm long and 0.45 ± 0.02 (0.29–0.51) mm wide. The anterior region exhibited a prominent boring tooth, and the lips were inconspicuous. The esophagus displayed an anterior muscular part measured 1.35 ± 0.02 (1.28–1.46) mm long. The ventriculus was long and cylindrical measured 0.78 ± 0.15 (0.59–0.98) mm long and 0.17 ± 0.02 (0.12–0.22) mm wide. The excretory pore is located anteriorly. The intestine was long. The anus is located at 0.95 ± 0.15 (1.12–1.45) mm from the posterior end. Also, scanning electron microscopy showed the characteristic triradiate mouth opening surrounded by four amphids and transverse and longitudinal striations of the cuticle (Figs. 1, 2, and 3). These characteristic features of L3 larvae were identical to *Anisakis* larvae type I, particularly the long ventriculus and mucron at the posterior larval end (Mattiucci et al. 2018; Shamsi 2021). These larvae also displayed short rounded tails with a characteristic cylindrical bently protruded mucron measuring 0.018 ± 0.002 (0.0162–0.027) mm long, much similar to previous descriptions of Tunya et al. (2020) and Hien et al. (2021). In addition, the present species was compared, as shown in Table 2, with other previously published *A*. *typica*. Most of its body measurements were closer to those described from the Asian region rather than from Egypt which confirming this specie’s worldwide distribution.

**Table 2:** Morphometric comparison (in mm) of the present *A. typica* with some previously published species

Species	*Anisakis typica*	*Anisakis typica*	*Anisakis typica*	*Anisakis typica*	*Anisakis typica* (present study)
Host	Atlantic mackerel, *Scomber scombrus*	*Nemipterus hexodon* and *N. japonicus*	Indian mackerel *Rastrelliger kanagurta*	Some marine fishes	*Plectropomus maculates*
Regional distribution	Egypt	The Gulf of Thailand	Chiang Mai, Thailand	Vietnam	Red Sea, Egypt
References	Mostafa et al. (2020)	Tunya et al. (2020)	Cheypanya et al. (2021)	Hien et al. (2021)	Present study
Total body length	14.87–22 mm	12.31–18.00	17.79 ± 1.86 (14.48–20.19)	18.6 ± 1.1 (17.2–20.3)	18.1 ± 2.1 (16.58–19.68)
Maximum body width	0.38–0.56 mm	0.34–0.45	0.46 ± 0.01 (0.44–0.48)	0.29 ± 0.03 (0.26––0.34)	0.45 ± 0.02 (0.29–0.51)
Ventriculus length	0.47–0.75 mm	0.55–0.94	0.64 ± 0.07 (0.50–0.68)	0.64 ± 0.04 (0.58–0.82)	0.78 ± 0.15 (0.59–0.98)
Ventriculus width	–––	0.10–0.20	0.20 ± 0.03 (0.15–0.25)	––-	0.17 ± 0.02 (0.12–0.22)
Mucron length	0.08–0.18 mm	0.01–0.02	0.020 ± 0.002 (0.018–0.023)	0.025 ± 0.005 (0.021–0.030)	0.018 ± 0.002 (0.0162–0.027)
Mucron shape	A small mucron	Terminal cylindrical mucron	Terminal cylindrical mucron	Terminal cylindrical mucron	Terminal cylindrical mucron
Esophagus length (anterior part)	1.78 ± 0.05 (1.302–2.098)	1.36–1.88 (1.58 ± 0.19)	1.59 ± 0.09 (1.50–1.73)	1.52 to 1.58 (1.54 ± 0.02)	1.35 ± 0.02 (1.28–1.46)

Although Tunya et al. (2020) stated that the protruded mucron of L3 larvae might be used to differentiate anisakid larvae at the species level, additional research is required to validate this identification. Various genes, including the internal transcribed spacer region (ITS), cytochrome oxidase subunits (cox1), and cox2 have been utilized to identify the nematode parasites (Zhang et al. 2018). The ITS1-5.8S-ITS2 region of ribosomal DNA is a suitable marker for nematode species identification (Abollo et al. 2003), as this region exhibits higher nucleotide sequence differences between species.

**Fig. 4:**
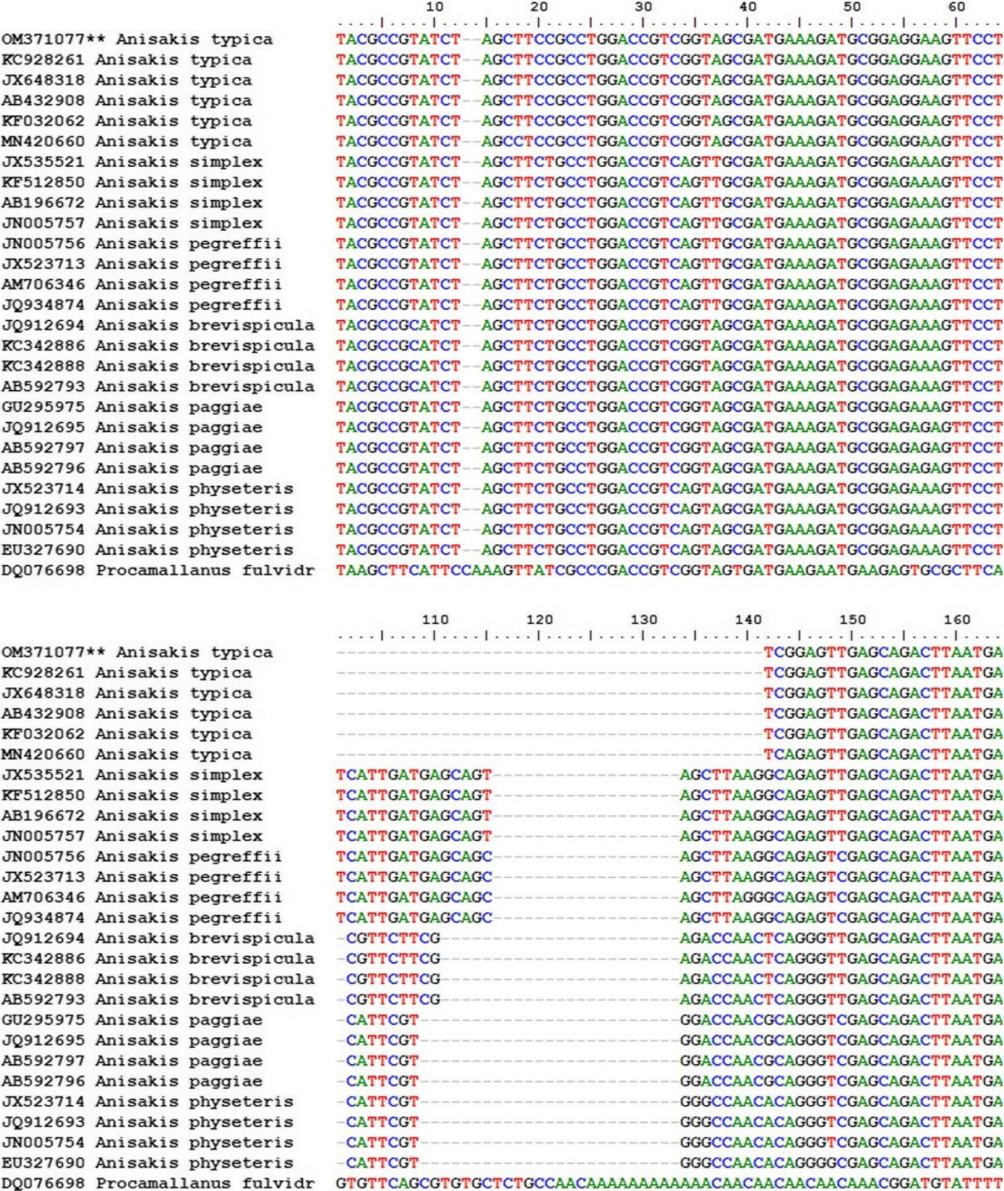
Multiple sequence alignment of ITS region of *Anisakis typica* larvae in the present study (accession number: OM371077 **) with some other *Anisakis* species reported in the Genbank using Bioedit (version 3.3.19.0)

**Fig. 5:**
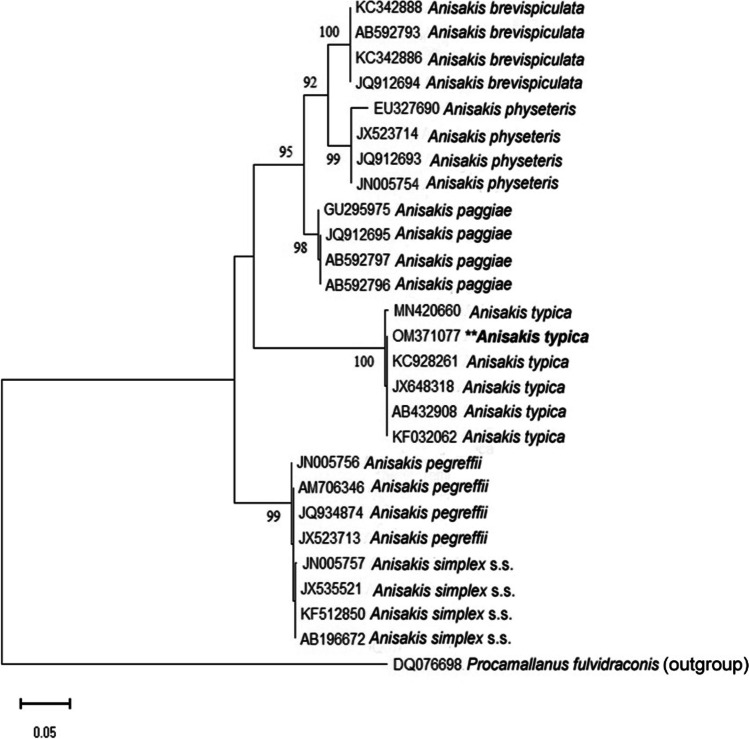
Maximum likelihood showing the phylogenetic relationships of *Anisakis typica* larva reported herein based on ITS of rDNA with other different species using Kimura 2-parameter model and 1000 bootstrap replications with a complete deletion. Bootstrap support values are indicated above the nodes, and *Procamallanus fulvidraconis* was used as an outgroup. Asterisks represent the present sample (OM371077 **)

**Fig. 6:**
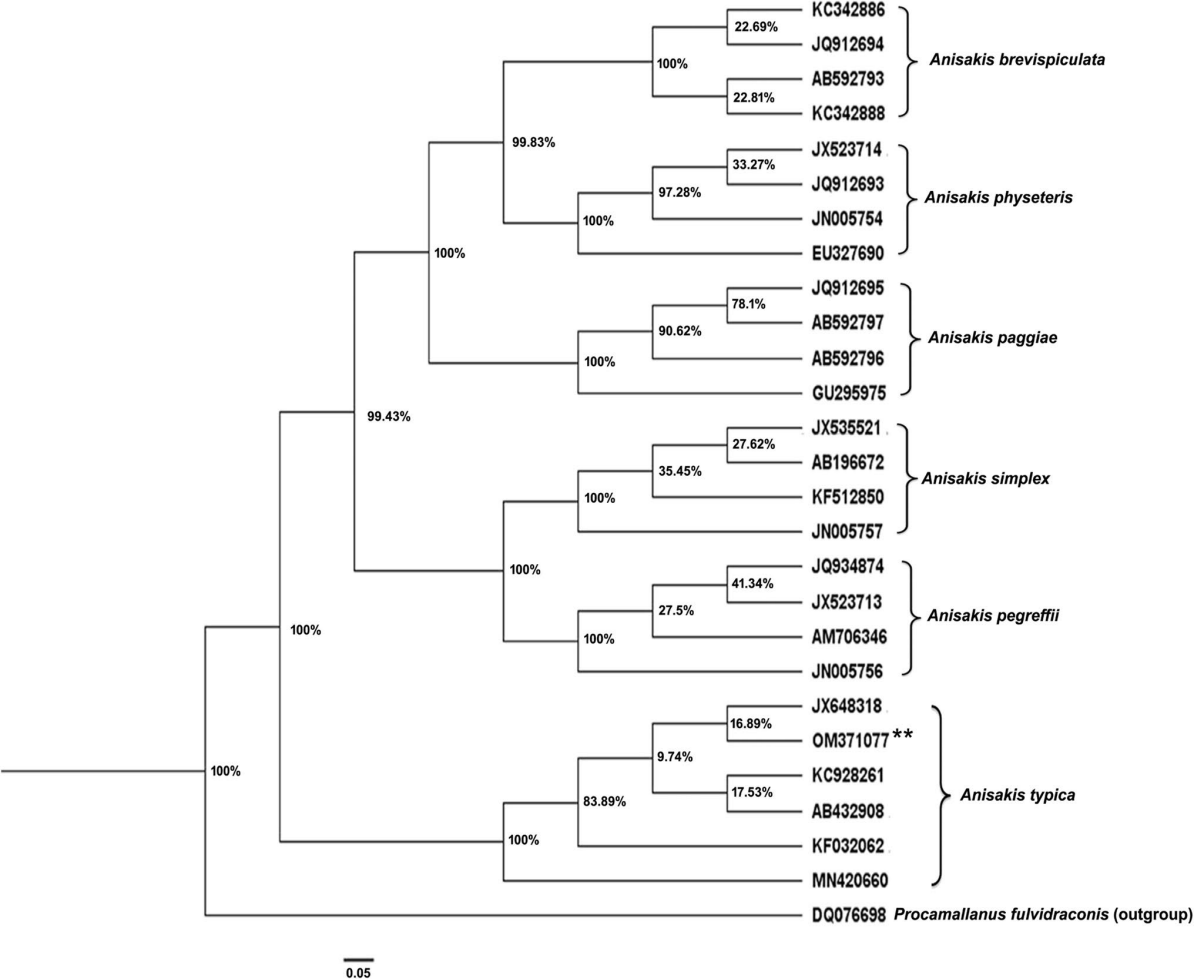
Phylogenetic tree was constructed based on ITS region of rDNA exploring the relationships among *Anisakis typica* of the present study using Bayesian inference (BI) method. Bootstrap percentages are shown at the internal nodes. *Procamallanus fulvidraconis* was used as an outgroup. Asterisks represent the present sample (OM371077 **)

Because the *Anisakis* larvae collected in this study were all morphologically similar, molecular identification was carried out on 25 isolated larvae. Nucleotide sequencing of the ITS region of rDNA (ITS-1, 5.8S, and ITS-2) yielded 670 bp and deposited in GenBank under the accession number OM371077. A comparison of *Anisakis* larval ITS1-5.8S-ITS2 rDNA nucleotide sequences exhibited high blast scores with previously published *A*. *typica* sequences in the GenBank, as shown in (Fig. 4). The greatest genetic similarity was 99.85% to specimens from Papua New Guinea (JX648318), Indonesia (KC928261), Coasts of Taiwan and Japan (AB432908), Turkish waters (KF032062), while 99.40% to (MN420660) from Thailand. Moreover, the phylogenetic trees of ITS regions of different *Anisakis* species were constructed using maximum likelihood and Bayesian methods as presented in (Figs. 5 and 6). The inferred topologies significantly supported the monophyly of *Anisakis* spp. The two phylogenetic methods produced similar topology results in terms of clades with high support values, confirming that our *Anisakis* larvae (OM371077) were clustered with other *A*. *typica* a distinct clade from other *Anisakis* species based on 100% bootstrap value explaining the genetic relationship with those published in the GenBank and promoting us to identify the present species parasitizing *P. areolatus* as *A*. *typica*.

The close genetic relationship of the current *A*. *typica* from Egyptian waters, the Red Sea, with different specimens from various geographical locations, may be attributed to the widespread dispersal of *A*. *typica* and the migration of its final hosts, Cetaceans, and its larvae infecting several marine fishes around the world (Shamsi et al. 2017). *Anisakis* type I, particularly *A. simplex* (s.s.), *A*. *pegreffii*, and *A*. *typica*, have been recovered from a wide variety of fish all over the world (Ayun et al. 2021), and *A*. *typica* has been commonly found in warmer temperate and tropical seas (Mattiucci et al. 2018), with some reports from the Mediterranean coast of Gabes city in Tunisia, Australia, New Caledonia, China, Vietnam, and from Thailand (Farjallah et al. 2008; Jabbar et al. 2012; Shamsi et al. 2018; Guo et al. 2020; Tunya et al. 2020; Cheypanya et al. 2021).

Anisakiasis in humans was most frequently linked to *A*. *simplex* s.s. and *A*. *pegreffii* (Aibinu et al. 2019). But, cases of infection by *A*. *typica* were rarely recorded, and due to the lack of information regarding the illness caused by their larvae (Tunya et al. 2020), the zoonotic effect may be underestimated (Umehara et al. 2010). Furthermore, the parasite was only identified as *Anisakis* larval type I in the majority of human reports (Bruschi and Dupouy-Camet 2014). While molecular techniques can accurately identify species of *Anisakis* larvae, medical professionals don't employ them to distinguish anisakid larvae from their patients (Rahmati et al. 2020). In addition, *Anisakis* larvae infecting humans are frequently damaged or fragmented upon removal, preventing any attempt for identification at the genus level (Mattiucci et al. 2013), and the diagnosis of allergic anisakidosis is primarily based on non-specific serology tests (Ubeira 2014). Therefore, the majority of diagnoses assumed the larvae to be *Anisakis simplex* s.s. This means that the possibility of human infection by other species of *Anisakis* cannot be completely ruled out, although the current species was considered a non-zoonotic parasite until now. The existence of these parasites in the edible portion/muscle of the fishes posed a relatively high risk to human health when consumed. In this study, we used molecular analysis to identify *Anisakis* larvae based on ITS1-5.8S-ITS2 rDNA nucleotide sequences as *A*. *typica* in the marine fish coral trout, *P*. *areolatus*, a new host record from the Red Sea in Egypt.

### Specimen deposition

Specimens were deposited in Zoology Department, Faculty of Science, Cairo University, Cairo, Egypt (Code No. P002).

## Conclusion

Based on our detailed description utilizing light and scanning electron microscopy and advanced molecular analysis such as DNA sequencing, we were able to elucidate the precise taxonomy of the current larvae as *A. typica*. This study adds important information to the previous reports on the occurrence of *Anisakis* larvae infecting commercial fish in Egypt, which may be highly relevant to public health issues, and highlights the necessity of further research on other fishes preferred by the Egyptian population.
