# Correction to: Morphological and molecular identification of third‑stage larvae of *Anisakis typica* (Nematoda: Anisakidae) from Red Sea coral trout, *Plectropomus areolatus*

**DOI:** 10.1007/s00436-023-07802-w

**Published:** 2023-03-03

**Authors:** Nesma Abbas Mostafa, Fathy Abdel‑Ghaffar, Hamed Omar Fayed, Ayat Adel Hassan

**Affiliations:** Zoology Department, Faculty of Science, Cairo University, Giza, Egypt

**Correction to** **:** **Parasitology Research**

10.1007/s00436-022-07776-1

The authors regret that there are some errors in their article, including the mis-identification of the fish host species.

The original article has been corrected.

